# Maternal SARS-CoV-2 infection during pregnancy: possible impact on the infant

**DOI:** 10.1007/s00431-021-04221-w

**Published:** 2021-08-05

**Authors:** Patrick Morhart, Christian Mardin, Manfred Rauh, Jörg Jüngert, Johanna Hammersen, Sven Kehl, Wolfgang Schuh, Sigrun Maier-Wohlfart, Katharina Hermes, Antje Neubert, Michael Schneider, Alexander Hein, Joachim Woelfle, Holm Schneider

**Affiliations:** 1 Department of Pediatrics, University of Erlangen-Nürnberg, Loschgestr. 15, 91054 Erlangen, Germany; 2 Department of Ophthalmology, University of Erlangen-Nürnberg, Erlangen, Germany; 3 Department of Obstetrics and Gynecology, University of Erlangen-Nürnberg, Erlangen, Germany; 4 Division of Molecular Immunology, University of Erlangen-Nürnberg, Erlangen, Germany; 5 Hauner’sches Kinderspital, University of Munich, Munich, Germany

**Keywords:** Coronavirus, Materno-fetal transmission, Embryopathy, Malformation, Case report

## Abstract

The risk and potential consequences of mother-to-child transmission of severe acute respiratory syndrome-coronavirus type 2 (SARS-CoV-2) during pregnancy are still a matter of debate. We studied the impact of SARS-CoV-2 infection on 56 complete households, including 27 newborns whose mothers were pregnant when exposed to the virus. Two PCR-confirmed perinatal SARS-CoV-2 transmissions with mild symptoms in affected neonates were recorded. In addition, we observed a severe eye malformation (unilateral microphthalmia, optic nerve hypoplasia, and congenital retinopathy) associated with maternal SARS-CoV-2 infection in weeks 5 and 6 of embryonic development. This embryopathy could not be explained by other infectious agents, genetic factors, drug use, or maternal disease during pregnancy. Eight other women with a history of SARS-CoV-2 infection prior to gestational week 12, however, delivered healthy infants.

*Conclusion*: The repeated occurrence of mother-to-child transmission in our cohort with risks that remain incompletely understood, such as long-term effects and the possibility of an embryopathy, should sensitize researchers and stimulate further studies as well as support COVID-19 vaccination recommendations for pregnant women.

*Trial registration number:* NCT04741412. Date of registration: November 18, 2020

**Table:** 

**What is Known:** *•Materno-fetal transmission of severe acute respiratory syndrome-coronavirus 2 (SARS-CoV-2) during pregnancy has rarely been reported so far, but was demonstrated in isolated cases.*	
**What is New:** *•In a study of complete households with documented SARS-CoV-2 infection, including a cohort of pregnant women, we observed perinatal coronavirus transmission at a higher frequency than expected.* *•We also describe a newborn boy with an eye malformation reminiscent of rubella embryopathy but associated with early gestation SARS-CoV-2 infection of his mother.* *•A coronavirus-related embryopathy, reported here for the first time, is a finding that requires further investigation.*

**Supplementary information:**

The online version contains supplementary material available at 10.1007/s00431-021-04221-w.

Maternal infections with severe acute respiratory syndrome-coronavirus 2 (SARS-CoV-2) during pregnancy usually do not have severe consequences for mother and child. As some pregnant women, however, become seriously ill with coronavirus disease of 2019 (COVID-19), the primary risk to the infant appears to arise from maternal illness [1]. Transmission of SARS-CoV-2 to the fetus is probably a very rare event [2, 3]. Therefore, possible complications of such pathogen spread are scarce and hard to capture. Potential harm to the fetus depends on many factors, particularly on the developmental stage of the unborn child at the time of viral infection.

We studied the impact of SARS-CoV-2 infection on 56 complete households, including 27 newborns whose mothers were pregnant when exposed to the virus. Among these infants, we recorded two unquestionable mother-to-child transmissions of SARS-CoV-2 shortly before or during delivery. In addition, the son of a woman with early gestation COVID-19 has an eye malformation similar to those observed after maternal rubella in the first trimester of pregnancy, an issue to be put up for discussion in the scientific community.

## Subjects and methods

### Study population

In a longitudinal study of 56 complete households (231 individuals) with one to eight members who had COVID-19 and/or developed antibodies against SARS-CoV-2, we investigated the course of disease, immune responses, and long-term consequences of the infection (ClinicalTrials.gov NCT04741412). This study was approved by the ethics committee of the University Erlangen-Nürnberg and conducted in accordance with the principles of the declaration of Helsinki. All individuals or their legal guardians provided written informed consent to participate. The cohort included 27 newborns of women who had acquired a SARS-CoV-2 infection during pregnancy. Clinical and sonographic follow-up examinations were performed in the first 5 weeks after birth.

### Diagnosis of SARS-CoV-2 infection

SARS-CoV-2 infection was diagnosed based on exposure history, clinical manifestation, and a positive reverse transcription-polymerase chain reaction (RT-PCR) result. Material from respiratory tract swabs, placental tissue, and cord blood samples were investigated. The qualitative cobas SARS-CoV-2 dual-target RT-PCR assay (SARS-CoV-2 specific target 1: ORF1/a region; pan-Sarbecovirus-specific target 2: envelope E region), running on an automated PCR system (cobas 6800; Roche Diagnostics, Mannheim, Germany), displays cycle threshold (Ct) values for both viral target sequences and the internal control. According to the manufacturer’s evaluation, the detection limit for SARS-CoV-2 RNA in respiratory tract swabs is 0.009 tissue culture infectious dose 50% (TCID50)/mL for target 1 and 0.003 TCID50/mL for target 2.

### Detection of specific antibodies

Patients’ sera were screened for anti-SARS-CoV-2 antibodies using the NADAL COVID-19 IgG/IgM test (nal von minden GmbH, Moers, Germany) or the Anti-SARS-CoV-2 ELISA (Euroimmun, Lübeck, Germany) according to the manufacturers’ instructions.

Flow cytometry–based assessments of antibodies against the SARS-CoV-2 spike protein were performed as described previously [4, 5]. In brief: A plasmid-encoding SARS-CoV-2 spike was co-transfected together with a green fluorescent protein (GFP)–encoding plasmid into HEK293T cells. Two days later, the cells were incubated with serum samples from patients (1:100 dilution), followed by staining with a secondary antibody mixture of PE-conjugated anti-human IgA (Southern Biotech, Birmingham, USA), AF647-conjugated anti-human IgG (Southern Biotech) and DyLight405-conjugated anti-human IgM (Jackson ImmunoResearch, Ely, UK) antibodies. Stained cell populations were analyzed using a Gallios flow cytometer (Beckman-Coulter). The antibody TRES 224 [5, 6] recognizing the SARS-CoV-2 spike protein served as positive control.

An electrochemiluminescence immunoassay to detect IgG specific for the SARS-CoV-2 nucleocapsid was performed by SYNLAB International GmbH (Weiden, Germany).

### DNA sequence analysis

After obtaining informed consent from the parents, genomic DNA of a newborn patient with an eye malformation was investigated, first by Sanger sequencing of the gene *PAX6*, variants of which are most frequently associated with optic nerve hypoplasia. Specific primers covering the exons and intron–exon boundaries were designed using the online design and analysis tool ExonPrimer (https://ihg.gsf.de/ihg/ExonPrimer.html) and the in-silico PCR tool from the University of California, Santa Cruz (https://genome.ucsc.edu/). Primer sequences and thermal cycling conditions are available upon request. DNA extraction, PCR, and sequencing were performed as described previously [7]. Secondly, a blood sample from this patient was sent to a provider of genetic diagnostics and sequencing services (CeGaT GmbH, Tübingen, Germany) for whole-exome sequencing using the Illumina NovaSeq6000 Sequencing Systems. The bioinformatic data obtained were analyzed with the Golden Helix GenomeBrowse tool (Golden Helix, Bozeman, USA). Our selection of potentially relevant genes was based on three gene panels published recently: the panel for ocular malformations from CeGaT (31 genes), a gene panel from DBGen-Ocular Genomics (76 genes; https://dbgen.com/en/test/neuro-ophthalmological-disorders-panel/), and a partially overlapping panel of 28 genes associated with aplasia/hypoplasia of the optic nerve from the HPO Gene-Disease Associations dataset (https://maayanlab.cloud/Harmonizome/gene_set/aplasia%24slash%24hypoplasia+of+the+optic+nerve/HPO+Gene-Disease+Associations), comprising a total of 121 different genes. Criteria for evaluating detected variants were for example population allele frequencies, genomic positions, predicted effects on biological function, and data published in the available scientific literature. Each potentially pathogenic variant identified in this study was assessed with the mutation prediction tools Mutation Taster (Charite, Berlin, DE; Cardiff University, Cardiff, UK) and the Ensembl Variant Effect Predictor also containing the SIFT and PolyPhen-2 scores for protein changes (European Molecular Biology Laboratory’s European Bioinformatics Institute, Hinxton, UK).

## Results and discussion

Clinical findings in our study population indicate a very low risk of severe COVID-19 in children and adolescents. More than half of the individuals below 18 years of age remained asymptomatic (Table 1). In 80% of the families investigated, an adult was the first household member with COVID-19 symptoms. Specific antibodies (IgG recognizing the SARS-CoV-2 spike protein) were detected in 69% of children and adolescents and in 88% of adults exposed to SARS-CoV-2 (Table 1). As serum samples could often be obtained only 6–12 weeks after the infection, the IgM response against SARS-CoV-2 was not detectable anymore in 49% of the subjects with specific IgG. Apart from a 15-year-old girl who was hospitalized because of a coincidence of COVID-19 with manifestation of type 1 diabetes mellitus, no subject below 18 years had to be hospitalized.

**Table 1:** Age-dependent manifestation of COVID-19 in households with confirmed SARS-CoV-2 infection

	**Adults**	**Adolescents, 13–17 years**	**Children, 0–12 years**	**Infants exposed pre- or perinatally**
Frequency of reported or clinically evident primary COVID-19 symptoms	104/121 (86.0%)	12/24 (50.0%)	24/59 (40.7%)	4/27 (14.8%)
First household member with COVID-19 symptoms	44/55* (80.0%)	5/55* (9.1%)	6/55* (10.9%)	0/55* (0.0%)
Subjects with specific antibodies (IgG against the SARS-CoV-2 spike protein)	107/121 (88.4%)	17/24 (70.8%)	40/59 (67.8%)	19/27 (70.4%)^#^

^*^In one of the 56 households enrolled in this study, nobody had COVID-19 symptoms, but all household members developed antibodies against SARS-CoV-2

^#^Serum of newborn infants was investigated within 3 days after birth; specific IgG detected in these samples were most likely maternal antibodies

In our cohort of pregnant women, nine symptomatic maternal SARS-CoV-2 infections occurred in the first trimester of pregnancy (one during gestational week 4, two in week 6, one in week 7, three in week 8, one in week 9, and one in week 11 after the last menstrual period). Six affected women developed moderate illness lasting several days, but none of them had to be hospitalized. However, one of these women delivered a boy (subject M12) with a severe eye malformation.

### Case report

**Fig. 1:**
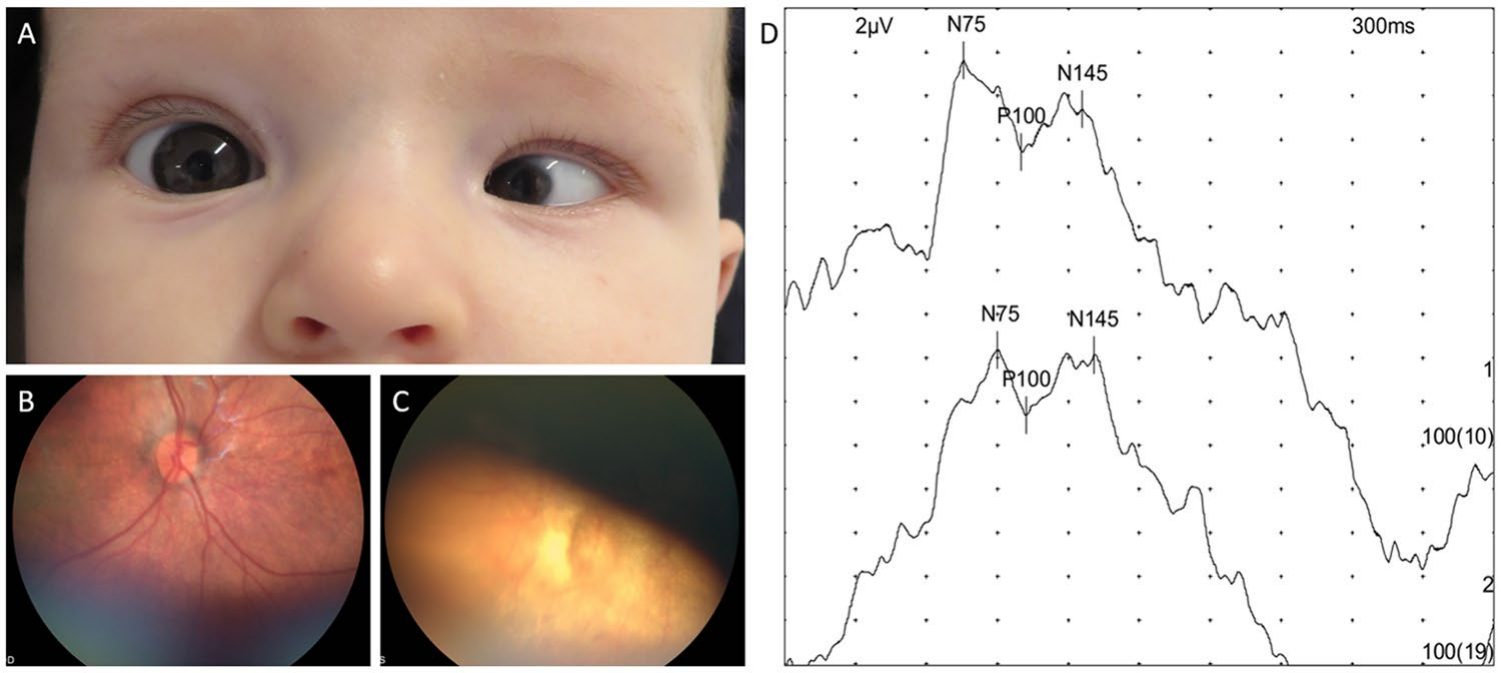
Malformation of the left eye associated with maternal SARS-CoV-2 infection during the first trimester of pregnancy. **A** Unilateral microphthalmia, microcornea, and persistent pupillary membrane in the newborn infant of a 40-year-old woman who had COVID-19 in gestational week 8. SARS-CoV-2 replication most likely occurred during Carnegie stages 15 and 16 of embryonic development*.* **B** Normal ocular fundus of the right eye, markedly different from that of the left eye (panel **C**) which is characterized by hypoplasia of the optic nerve, neurosensory retina, and retinal pigment epithelium, and by missing retinal vasculature. **D** Normal visually evoked potentials under binocular flash stimulation (P100 = 100.31 and 102.19 ms)

The mother of subject M12 had shown typical COVID-19 symptoms (fever, strong headache, nausea, fatigue, loss of smell and taste) from day 54 after the last menstrual period (day 40 after conception) for 1 week, followed by an otherwise uneventful pregnancy. Her newborn son was later diagnosed with unilateral microphthalmia, microcornea, and hypoplasia of both the optic nerve and the neurosensory retina (Fig. 1A–C). Similar eye abnormalities, uni- or bilaterally, are seen in neonates with rubella embryopathy. The patient’s second eye and visually evoked potentials under flash stimulation (Fig. 1D) were found to be normal. Any relevant family history as well as rubella, toxoplasmosis, herpes, or cytomegalovirus infection have been excluded; vaccination of the mother against rubella before becoming pregnant and protective levels of rubella-specific IgG were evident. As the father had contracted COVID-19 5 days before his wife, SARS-CoV-2 replication in the mother most likely happened in weeks 5 and 6 of embryonic development, during oculogenesis [8]. Maternal PCR testing for SARS-CoV-2 (nasopharyngeal swab) 3 days after symptom onset was positive. Both parents developed specific antibodies against the SARS-CoV-2 spike protein. At birth in gestational week 41 + 1, the infant weighed 4000 g; SARS-CoV-2 PCR tests were negative, while IgG against the nucleocapsid of SARS-CoV-2 (most likely of maternal origin) were still detectable in the cord blood. Ultrasonography of the brain and abdomen did not reveal additional malformations. Five months after birth no antibodies against SARS-CoV-2 could be detected anymore in the infant’s serum.

We attempted to rule out genetic causes of optic nerve hypoplasia in this patient by whole-exome sequencing and careful analysis of 121 genes possibly involved in developmental anomalies of the eye (three complete gene panels for ocular malformations, including *ABCB6*, *BMP4*, *GDF3*, *GDF6*, *MCAT*, *PAX2*, *PAX6*, *POMT1*, *RAX*, *SOX2*, *SOX3*, *TMEM98*, and *WNT3*). We detected a total of three gene variants with an allele frequency below 1%: The first of them, heterozygous *ABCB6* variant c.649C > A (p.Q217K; transcript CCDS2436.1; ENST00000265316; NM_005689.4), was classified as benign by each of the mutation prediction tools used. It affects an exon in one of the two transcripts produced but an intron in the other transcript. The second variant of uncertain significance, heterozygous *RAX* variant c.311G > A (p.G104D; transcript ENST00000256852.7) which is identical with c.565G > A (p.A189T) in another *RAX* transcript (CCDS11972.1; ENST00000334889; NM_013435.3), does not, in our opinion, explain the phenotype, because *RAX* variants have been considered as pathogenic in the literature only when present homozygously or compound-heterozygously. The third finding of potential significance, heterozygous *MCAT* variant c.235C > G (p.R79G; transcript CCDS14045.1; ENST00000327555; NM_014507.3), was classified as benign based on the SIFT and PolyPhen-2 scores and has been detected more frequently in a Swedish subpopulation. However, in order to definitely rule out a genetic origin of subject M12’s eye malformation, a whole-genome analysis would be needed.

Known teratogenic factors that may influence optic nerve development, such as maternal alcohol consumption during early gestation, smoking, drug abuse, and maternal diabetes mellitus, were also excluded. As the patient’s mother did not take any potentially teratogenic drugs, our findings suggest the eye abnormalities of the infant to be due either to materno-fetal transmission of SARS-CoV-2 or to an indirect effect of the maternal SARS-CoV-2 infection at the time of optic tract development. This conclusion must be taken with caution, because the only link that connects the association is the fact that the patient’s mother contracted COVID-19 during the relevant stage of embryogenesis. Given the high incidence of SARS-CoV-2 infection, the link may be entirely coincidental. On the other hand, such findings have to be reported to the scientific community to allow further investigation and to raise awareness of rare complications associated with COVID-19.

The other eight newborns of mothers with symptomatic SARS-CoV-2 infection during early gestation did not show any clinical or ultrasonographic abnormality within 3 months after birth.

Although in 23 out of 27 neonates investigated in this study, no morbidity related to preceding SARS-CoV-2 infection of their mothers was observed, maternal infection at the very end of pregnancy (*n* = 5), before sufficient passive immunity could be conferred to the baby, led to positive SARS-CoV-2 PCR results and mild COVID-19 symptoms in two newborns (40%; one male, one female), including a generalized postinfectious exanthema (Supplementary Fig. 1A) that lasted for up to 14 days. An almost identical skin rash was observed with a 3-day delay also in the otherwise healthy, postnatally SARS-CoV-2 PCR-negative dizygotic twin of the affected girl.

In the meantime, the clinical spectrum of neonatal COVID-19 has been reviewed systematically [9] and the World Health Organization issued an official definition (https://www.who.int/publications/i/item/WHO-2019-nCoV-mother-to-child-transmission-2021.1). Recognition of potential cutaneous manifestations, however, is still challenging. Differential diagnosis in newborn patients during a pandemic requires a particularly careful assessment of clinical abnormalities. In one of our two symptomatic newborns, for example, clearly elevated but continuously decreasing D-dimers were recorded (Supplementary Fig. 1B), explainable by an obvious cephalohematoma rather than by COVID-19-related thrombophilia.

Sonography of the brain and adrenal glands of the five perinatally exposed infants did not reveal any additional abnormalities.

The comparatively mild course of disease in our pediatric subpopulation confirms what has been observed by others [10], although issues such as long COVID or myalgic encephalomyelitis/chronic fatigue syndrome [11] have not yet been investigated sufficiently. In addition, the data were obtained prior to the occurrence of SARS-CoV-2 variants of concern which appear to be transmitted more easily [12]. Perinatal SARS-CoV-2 infections of the infant leading to a mild course of disease have been reported elsewhere, too [1, 9], although their frequency in our study was higher than expected. An embryopathy associated with maternal COVID-19 is described here for the first time. In order to identify the pathogenetic mechanisms underlying a link between SARS-CoV-2 infection and ocular developmental anomalies, further studies involving animal experiments would be required. Even if a direct impact of the virus on neurodevelopment was considered unlikely, cytokine storm and hyperinflammatory processes in a pregnant woman with SARS-CoV-2 infection could still increase the risk of neurodevelopmental disorders in early stages of pregnancy [13]. Based on the biological properties of SARS-CoV-2, Leyser and colleagues speculate that the virus might neutralize the maternal immune response by affecting interferon type 1 expression, similar to Zika virus, thereby facilitating virus spread and increasing the risk of damage to progenitor cells of the fetal brain [13].

The possibility of an embryopathy induced by SARS-CoV-2 infection during pregnancy should sensitize researchers and stimulate more systematic studies. If further cases of potentially coronavirus-related malformations become known, COVID-19 vaccination recommendations for pregnant women may need to be encouraged.

## Supplementary Information

Below is the link to the electronic supplementary material.

Supplementary file1 (PPTX 451 KB)

## Data Availability

The datasets used and analyzed during the study are available from the corresponding author upon reasonable request.

## Abbreviations

SARS-CoV-2: Severe acute respiratory syndrome-coronavirus 2
COVID-19: Coronavirus disease of 2019
(RT-)PCR: (Reverse transcription-)polymerase chain reaction
GFP: Green fluorescent protein

## References

[1] Adhikari EH, MorenoW ZAC, MacDonald L, McIntire DD, Collins RRJ (2020). Pregnancy outcomes among women with and without severe acute respiratory syndrome coronavirus 2 infection. JAMA Netw Open.

[2] Vivanti AJ, Vaoloup-Fellous C, Prevot S, Zupan V, Suffee C, Do Cao J (2020). Transplacental transmission of SARS-CoV-2 infection. Nat Commun.

[3] Fenizia C, Biasin M, Cetin I, Vergani P, Mileto D, Spinillo A (2020). Analysis of SARS-CoV-2 vertical transmission during pregnancy. Nat Commun.

[4] Lapuente D, Maier C, Irrgang P, Hübner J, Peter AS, Hoffmann M (2021). Rapid response flow cytometric assay for the detection of antibody responses to SARS-CoV-2. Eur J Clin Microbiol Infect Dis.

[5] Schuh W, Baus L, Steinmetz T, Schulz SR, Weckwerth L, Roth E et al (2021) A surrogate cell-based SARS-CoV-2 spike blocking assay (SUBA). Eur J Immunol, under review 10.1002/eji.202149302 PMC8646767 34547822

[6] Peter AS, Roth E, Schulz SR, Fraedrich K, Steinmetz T, Damm D et al (2021) A pair of non-competing neutralizing human monoclonal antibodies protecting from disease in a SARS-CoV-2 infection model. https://www.biorxiv.org/content/10.1101/2021.04.16.440101v1 (Preprint) 10.1002/eji.202149374 PMC8420377 34355795

[7] Wohlfart S, Schneider H (2019). Variants of the ectodysplasin A1 receptor gene underlying homozygous cases of autosomal recessive hypohidrotic ectodermal dysplasia. Clin Genet.

[8] Harding P, Moosajee M (2019). The molecular basis of human anophthalmia and microphthalmia. J Dev Biol.

[9] Raschetti R, Vivanti AJ, Vauloup-Fellous C, Loi B, Benachi A, De Luca D (2020). Synthesis and systematic review of reported neonatal SARS-CoV-2 infections. Nat Commun.

[10] Götzinger F, Santiago-Garcia B, Noguera-Julian A, Lanaspa M, Lancella L, Carducci FIC (2020). COVID-19 in children and adolescents in Europe: a multinational, multicentre cohort study. Lancet Child Adolesc Health.

[11] Couzin-Frankel J (2020). The long haul. Science.

[12] Plante JA, Mitchell BM, Plante KS, Debbink K, Weaver SC, Menachery VD (2021). The variant gambit: COVID-19's next move. Cell Host Microbe.

[13] Leyser M, Pinto Marques FJ, Moreira de Nascimento OJ (2021). Potential risk of brain damage and poor developmental outcomes in children prenatally exposed to SARS-CoV-2: A systematic review. Intern Paul Pediatr.

