# Supplementary figures and images for: Maternal SARS-CoV-2 infection during pregnancy: possible impact on the infant
Source: Eur J Pediatr. 2021 Aug 5;181(1):413–8. doi: 10.1007/s00431-021-04221-w (PMC8341836; doi:10.1007/s00431-021-04221-w)

## Slide 1
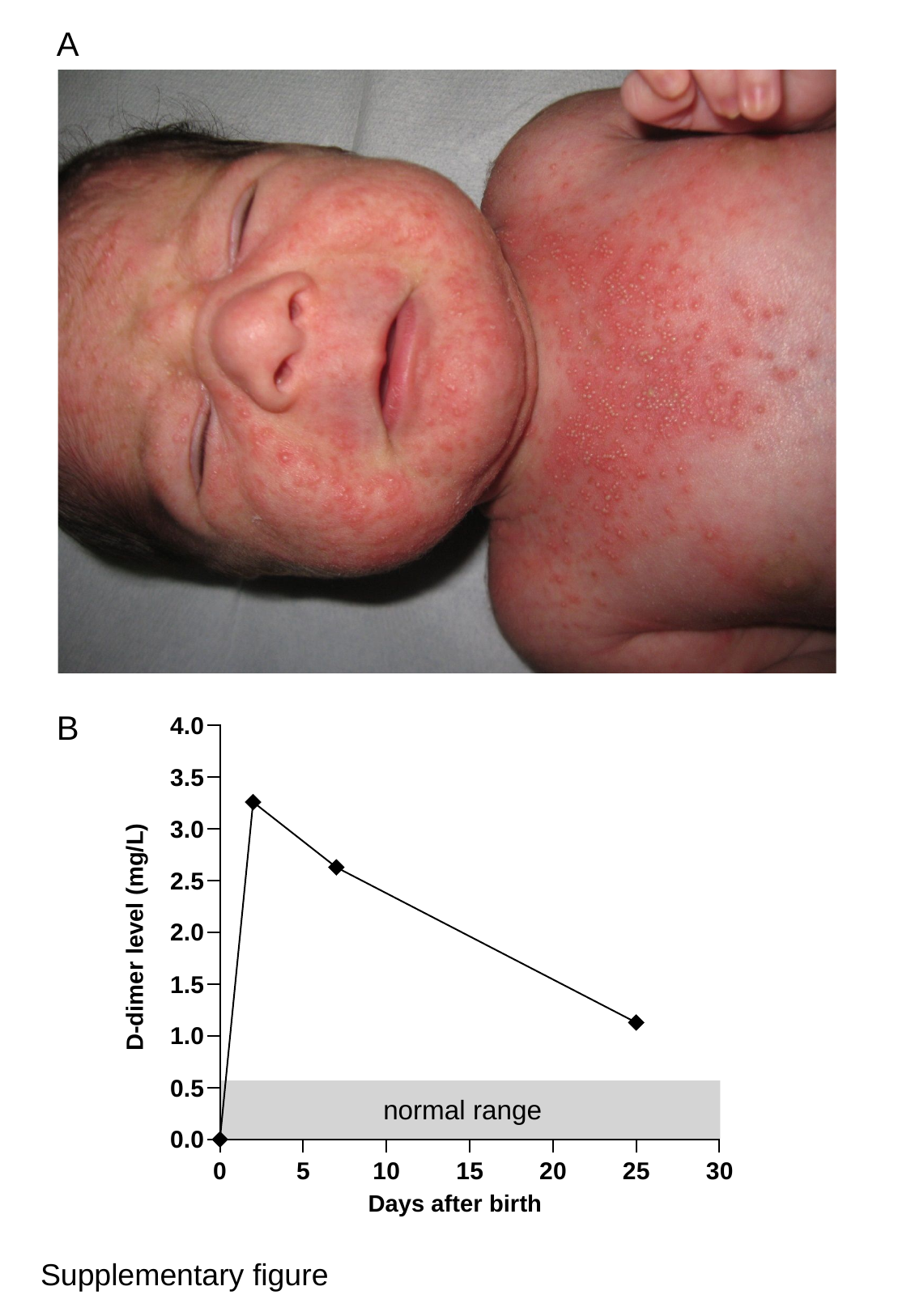

A
B
D-dimer level (mg/L)
normal range
Days after birth
Supplementary figure

Supplement: Supplementary file 1 — Supplementary file1 (PPTX 451 KB) [file 431_2021_4221_MOESM1_ESM.pptx]
